# Clinical Impact of Preprocedural CT-Based 3D Computational Simulation of Left Atrial Appendage Occlusion with Amulet

**DOI:** 10.1155/2021/9972228

**Published:** 2021-07-26

**Authors:** Ian Buysschaert, Dries Viaene

**Affiliations:** ^1^AZ Sint-Jan Brugge-Oostende AV, Ruddershove 10, Brugge 8000, Belgium; ^2^ASZ, Merestraat 80, Aalst 9300, Belgium

## Abstract

**Aims:**

Standard of care (SoC) device size selection with transoesophageal echocardiography (TOE) and computed tomography (CT) in LAAO can be challenging due to a certain degree of variability at both patient and device levels. The aim of this study was to prospectively evaluate the clinical impact of 3D computational modelling software in the decision-making of left atrial appendage occlusion (LAAO) with Amplatzer Amulet.

**Methods and Results:**

SoC preprocedural assessments as well as CT-based 3D computational simulations (FEops) were performed in 15 consecutive patients scheduled for LAAO with Amulet. Preprocedural device size selection and degree of confidence were determined after SoC and after FEops-based assessments and compared to the implanted device. FEops-based preprocedural assessment correctly selected the implanted device size in 11 out of 15 patients (73.3%), compared to 7 patients (46.7%) for SoC-based assessment. In 4 patients (26.7%), FEops induced a change in device size initially selected by SoC. In the 7 patients (46.7%) in which FEops confirmed the SoC device size selection, the degree of confidence of the size selection increased from 6.4 ± 1.4 for SoC to 8.1 ± 0.7 for FEops. One patient (6.7%) could not be implanted for anatomical reason, as correctly identified by FEops.

**Conclusions:**

Preprocedural 3D computational simulation by FEops impacts Amulet size selection in LAAO compared to TOE and CT-based SoC assessment. Operators could consider FEops computational simulation in their preprocedural device size selection.

## 1. Introduction

Left atrial appendage (LAA) occlusion is a well-accepted alternative to oral anticoagulant in the prevention of stroke or systemic thromboembolism in patients with atrial fibrillation (AF) [1]. While the occlusion is effective, the procedure can sometimes be challenging.

Part of the challenge is based on the high degree of variability, both at the patient level (anatomy, size, rhythm, filling pressures, etc.) and at the device level (platform, size, depth, degree of conformity, etc.). Additional challenges might derive from information overload from multimodality imaging (2D or 3D transoesophageal echocardiography, computed tomography, plain angiography, intracardiac echo, etc.) and slow democratic decision-making process of large implanting teams (operators, echocardiographers, product specialists, fellows, nursing staff, etc). All these challenges are reflected in the 6.1% to 38% change in device sizes during implantation [2–4], inability to close the LAA in 1–2.7% up to 4.4% of the cases [2–4], procedural complications, lengthy procedures, and so forth. All these numbers are perhaps even higher in unpublished series and early-operator learning curves.

A well-prepared preprocedural planning is therefore essential. Simulation technology such as 3D printing or computational *in silico* modelling offers the tactile and/or visual evaluation of the exact left atrial appendage anatomy with the different devices *in situ*, allowing diligent preprocedural device size selection and adequate positioning. FEops HEARTguide™ is a CE-marked and commercially available computational tomography- (CT-) based simulation technology suite, offering physicians detailed preoperative insights into the interaction between the closure devices and the patient's left atrial appendage anatomy [5].

The clinical impact of simulation software in clinical decision-making in LAAO is yet unknown. The present study aims to investigate to what extent the FEops HEARTguide computational modelling tool influences standard of care preprocedural device size selection.

## 2. Methods

### 2.1. Patients

From January until October 2020, 15 consecutive patients with CT scan and FEops 3D computational simulation scheduled for LAAO with Amplatzer™ Amulet™ (Abbott, USA) were enrolled in this single-centre prospective study. At least two days before the procedure, patients were scheduled for preprocedural CT scan (Aquilion One, Canon Medical CT systems, Japan) and 3D transoesophageal echocardiography (TOE) (EPIC 7C, Philips, Netherlands) on the same morning, after an overnight fast and after intravenous fluid administration of 500 ml NaCl 0.9% over 2 hours before imaging. CT scans were uploaded into the commercially available FEops HEARTguide portal (FEops, Belgium) and 3D simulations were performed as previously described [5]. All 3D computational simulations were performed independently by FEops, without interaction with the implanting team. Due to Covid-19, some patients did not undergo preprocedural TOE but only CT scan.

### 2.2. Preprocedural Assessment

Preprocedural analyses of the images were done just before each procedure. TOE images were reviewed, and if no prior TOE was available, *ad hoc* TOE was performed after general anaesthesia induction but just before start of the procedure. CT scans were analysed by multiplanar reconstruction (MPR) using Vue PACS (version 12.1.6.1005, Carestream Health, USA). Based on these measurements, being our standard of care (SoC), the team came to a first Amulet size decision (Decision SoC) and was asked to ascertain a degree of confidence towards this first choice, on a scale from 1 to 10, with 10 representing the highest degree of confidence (Confidence SoC).

**Figure 1:**
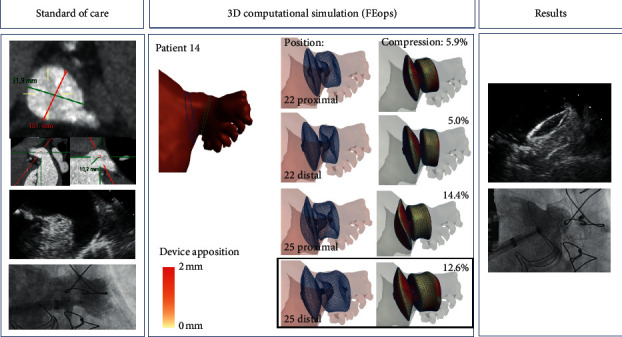
Illustrative case of a 70-year-old man with paroxysmal atrial fibrillation, CHADS-VASc score of 4, HAS-BLED score of 4, and previous intracranial haemorrhage scheduled for LAAO. Measurements of the os and landing zone, respectively, by 3D TEE were 23 × 31 mm and 19 × 22 mm, and those by CT were 22 × 30 mm and 19 × 22 (18.6 × 21.9 mm, left panel). Standard of care assessment had chosen for a 25 Amulet, with team's confidence 6 out of 10. FEops 3D rendering volume showed a chicken wing morphology (middle panel). Computation simulation of a 22 Amulet revealed limited compression (5.0 to 5.9%) and apposition both in proximal and distal positions, while a 25 Amulet had a better compression (12.6 to 14.4%) and apposition (see colour code, green 0 mm distance between device and appendage and red 2 mm) with good sealing of the os. A 25 Amulet with distal position was chosen with confidence 8 out of 10. Postimplantation images showed perfect position and sealing (right panel).

The results of the FEops 3D simulations were only accessed for the first time after Decision SoC and Confidence SoC were made by the implanting team, through the FEops portal (https://heartguide.feops.com/login). The general anatomy and orientation of the LAA were reviewed, followed by the 3D simulations of the different Amulet devices and their implantation depth. Based on all the available information, the team came to a second Amulet size decision (Decision FEops) and ascertained again a degree of confidence with regard to this second decision (Confidence FEops). See Figure 1 for an illustrative case example.

### 2.3. Procedure

All procedures were performed under general anaesthesia with cardiac angiography (Allura XPer FD10, Philips Healthcare, Netherlands) and TOE guidance (EPIQ 7C, Philips), unless asked otherwise by the patient (one patient asked for local anaesthesia and use of intracardiac echocardiography [ICE]). After transseptal puncture under echo guidance, the LAA was approached with the delivery sheath (TorqVue™ 45-45°, Abbott, USA) and protruding graduated 5F pigtail, followed by a contrast injection in one or two incidences for additional angiographic measurements. A final decision was done followed by implantation of an Amulet. Device compression was calculated on echocardiography, as well as residual leak. Technical success was defined as successful implantation of the device, and device success as technical success with no large leak (>3 mm).

### 2.4. Informed Consent

This registry was approved by the ASZ ethical committee, and all patients provided written informed consent. This study was an investigator-initiated study.

### 2.5. Statistical Analysis

Continuous variables are reported as mean ± SD and categorical variables as counts (percentages). *t*-test and Chi-square tests were used as appropriate.

## 3. Results

### 3.1. Baseline Characteristics

Baseline characteristics are shown in Table 1. A total of 15 patients were included, with a mean age of 77.5 ± 6.5 year, a CHA_2_DS_2_-VASc score of 4.7 ± 1.0, and HASBLED score of 3.1 ± 0.7, with varying indications ranging from bleeding disorders to recurrent stroke under oral anticoagulation.

### 3.2. Clinical Impact in Preprocedural Sizing

**Figure 2:**
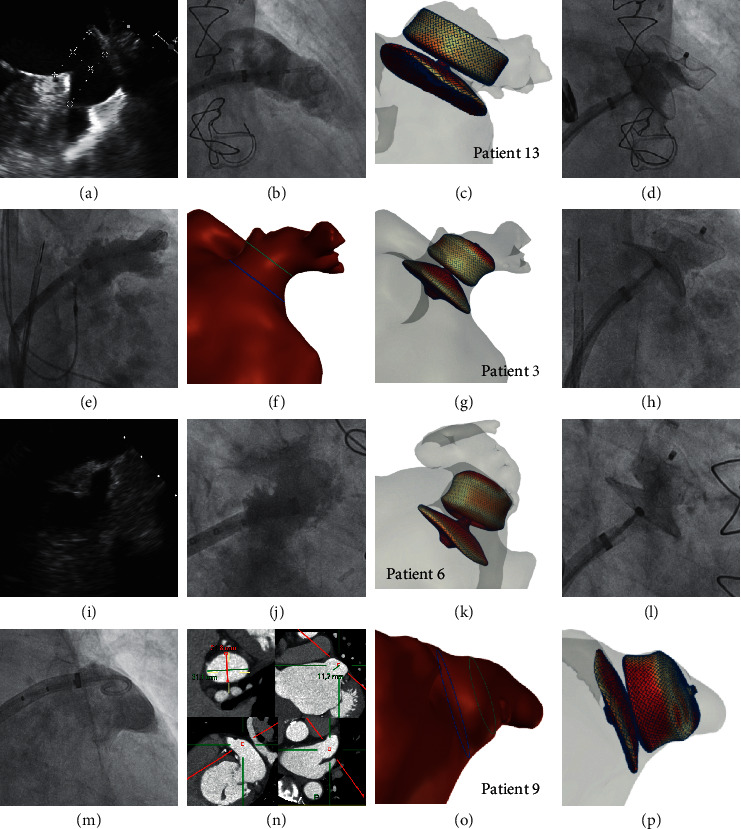
Case examples influenced by FEops. (a–d): patient 13, with a very large appendage, with mean landing zone of 31 mm on TOE (a), and chicken wing morphology with poor confidence (6 out of 10) for a 34 Amulet. FEops (c) confirmed good anchoring and sealing with a 34, with good angiographic results (d). (e–h): patient 3, implanted under local anaesthesia with intracardiac echocardiography. Windsock morphology on angiography (e) and 3D volume (f). Although initial assessment with CT (landing zone 16 × 18 mm) decided for a 20 mm, simulation with a 22 Amulet was preferred (g) with good angiographic results (h). (i–l): patient 6, reverse chicken wing morphology on TOE (i) and angiography (j), with an initial 22 Amulet selected based on TOE (3D landing zone 18 × 21 mm) and CT (landing zone 14 × 21 mm), but with a preferred 20 Amulet simulation (k) and good angiographic results once implanted (l). (m–p): patient 9, windsock with large os and almost no depth on angiography (m) and 3D volume (o), with landing zone 22 × 31 mm on CT (n); a 31 mm Amulet could not be implanted after multiple attempts. Note the absence of apposition (red) posterosuperior on the FEops simulation (p).

In 7 out of 15 cases (46.7%), the size selection with standard of care (Decision SoC) was similar to the size selection after analysis of the 3D computational simulation (Decision FEops) and was also the implanted device, while the confidence of the decision significantly increased from 6.4 ± 1.4 to 8.1 ± 0.7 (*p*=0.003) (Table 2). For example, in patient 13, the standard of care measurements demonstrated a very large appendage with chicken wing morphology (landing zone by TOE mean 31 mm, CT 21 by 36 mm), with uncertainty as to whether the largest 34 Amulet would anchor (hence confidence 6). Upon inspection of the FEops simulation, we had more confidence that a 34 would be feasible (confidence 8), confirmed by the implantation in sandwich and complete sealing of the LAA (Figure 2).

In 4 cases (26.7%), the initial size decision after standard of care (Decision SoC) was changed after analysis of the FEops simulation (Decision FEops), and the later correctly selected the implanted device. This implicates that the size selection based on the FEops simulation (Decision FEops) correlated better with the final implanted device, that is, 11 out of 15 cases (73.3%), compared to 7 out of 15 (46.7%) for the size selection based on standard of care alone (Decision SoC). In patient 6, for example (Figure 2), the landing zone on TOE was measured as 14 × 21 mm and on CT 14 × 21 mm. Based on these measurements, we had chosen a 22 Amulet with confidence 7. It was realised when using the FEops simulation that a 22 Amulet would be oversized and that a 20 device would be a better option, as confirmed by the device success of the 20 Amulet.

In 2 cases (13.3%), although both decisions based on the SoC measurements and FEops analyses were similar, it was decided to implant a different size during the implantation. In another case (patient 15), both the analyses based on SoC and FEops oversized the device. In another case (patient 9) a 31 device was selected both after standard of care and FEops, however, the device could not be implanted (see Figure 2 and narrative below).

### 3.3. Procedural Characteristics

All procedures were done under general anaesthesia with TOE guidance, except one under local anaesthesia with intracardiac echocardiography (ICE), upon request of the patient (Figure 2, patient 12). Mean fluoroscopy time and radiation exposure are presented in Table 3, as well as the implanted Amulet size and antithrombotic medication at discharge. One procedure was done with embolic protection device (Sentinel, Boston Scientific, USA) in a patient with a recent thrombus in the left atrial appendage and acute severe gastrointestinal bleeding precluding further anticoagulation. An Amulet device was successfully deployed in all but one patient, with no residual leak in all implanted patients (*n* = 14, device success 93.3%). There was no guide catheter or device size exchange (mis-sizing). In one patient, a 31 mm Amulet device could not be anchored safely at the posterior-superior wall of the appendage, due to an acute conical and tapered anatomy (Figure 2, patient 9). Several attempts were done, but the device was easily dislodged at every slight tug test. The 3D simulation indeed showed no apposition at the posterior-superior wall. The procedure was interrupted and the patient was further treated with warfarin. He had no recurrent stroke at 6 months' follow-up.

There were no periprocedural complications, in particular, no access site bleeding, cardiac perforation, device embolization, stroke, myocardial infarction, or death.

### 3.4. Follow-Up

None of the patients had any stroke, embolization, or other late complications at the latest follow-up (mean 144 ± 80 days).

## 4. Discussion

Our study is the first to demonstrate the clinical impact of FEops 3D computational simulation in preprocedural sizing in LAAO with Amulet. In a total of 15 consecutive and unselected patients planned for LAAO, preprocedural sizing based on standard of care and FEops correctly selected the finally implanted Amulet size in 11 patients (73.3%), compared to 7 (46.7%) after standard of care sizing alone. In 4 patients (26.7%), FEops-based sizing correctly induced a change in Amulet size compared to standard of care sizing, and in the 7 patients (46.7%) in which FEops confirmed the standard of care sizing, the team's confidence increased from 6.4 ± 1.4 to 8.1 ± 0.7. One patient could not be implanted due to challenging anatomy with insufficient anchoring of the Amulet. The FEops 3D simulation correctly showed lack of apposition at the posterior-superior wall, but the team decided to give it a try, which was unsuccessful. In retrospect, it might have been a better idea to perform a second simulation with another device architecture.

Although this first single-centre experience warrants further multicentre confirmation, we believe that the FEops 3D computation modelling is of value in the preprocedural sizing in LAAO. Given the anatomical complexity and interindividual variability of the LAA, 2D imaging modalities often fail to appreciate the exact morphology of the appendage due to incomplete spatial visualization. The 3D volume rendering of FEops quickly provides a general appraisal of the morphology and orientation of the appendage, while it also provides adequate measurements of both the OS and the landing zone. Consecutive sizes are simulated in different positions (proximal and distal), with two different modalities (frame deformation and device apposition), in conjunction with measurements of the device mid lobe diameters, offering the implanting team an overview of the best size and position to choose from, while being able to turn the appendage with implanted device in all possible directions. No suggestions are being made by the software with regard to the best device size. It remains the choice of the team to select the most appropriate size and position, based on the different simulations provided. While different positions are proposed (proximal and distal), further experience is warranted so as to determine whether these simulated positions and depths can always be achieved in practice. On a personal level, we strive for a thorough anchoring, with thereby a favour for a distal implantation whenever possible so as to avoid any eventual embolization.

TOE is currently the most used imaging modality to assess the anatomy and to perform the measurements of the LAA, since it is also the most often used for procedural guidance. However, TOE is known to underestimate the diameters of the landing zone [7], while CT provides the largest and most reliable LAA diameters to guide optimal device selection [6]. It has therefore been recommended to oversize with 3 to 6 mm the maximum diameter measured by 2D TOE for device selection, while an upsize of 2 to 5 mm above the mean CT LAA diameter is recommended [6]. This multimodality evaluation can sometimes result in two different size selections, for which the only solution is often to start the procedure and try one particular device size first. This can result in a higher degree of mis-sizing, longer instrumentation in the left atrium, and higher risk for complications. The use of FEops 3D computational simulation could be a better solution in those situations where multimodality evaluation is inconsistent.

Other simulation techniques for LAAO simulation are available, such as 3D printing [8]. While this technique offers more tactile feedback, it is not feasible for routine use in busy daily practice due to more complex logistics (courier delivery) and the need for additional bench testing with the different device sizes, in comparison to the server-based instant delivery and user-friendly interface of FEops computational modelling. We believe the latter is the most user- and resource-friendly.

We see a potential clinical benefit for the FEops platform, on a general level, for early-operators during their learning curves, for smaller centres performing ±20 procedures a year, or for first experiences with a novel device in an emerging market. Some particular settings might also benefit from FEops 3D computational modelling. For instance, in current Covid-19 pandemic times, TOE is a seen as a high-risk procedure that should be deferred whenever possible [9], implicating that preprocedural sizing with TOE might be replaced by CT, which can initially be challenging for centres with limited experience. The same holds true for procedures under local anaesthesia with the use of intracardiac echography (ICE) for procedural guidance instead of TOE. While ICE is sufficient for procedural guidance, it is certainly insufficient for precise measurements of the LAAO in terms of sizing [10]. Here again, CT-based preprocedural measurements, complemented with FEops 3D computational simulation, offer a valid alternative to TOE. FEops simulation also offers benefit when there is doubt about the feasibility of the procedure, for instance, in case the LAA anatomy is too large, too small, or too challenging for closure.

## 5. Study Limitations

Some limitations of this study should be acknowledged. One potential limitation of the FEops 3D computational modelling is the need for high-quality CT with appropriate use of iv contrast, which can be an issue in patients with severe kidney insufficiency. In addition, the size of the left atrial appendage is known to be filling-pressure-dependent [11]. It is therefore of major importance that the patient receives adequate iv fluids and is at least euvolemic during image acquisition.

This is the first published study of a single-centre experience with small sample size, and further prospective trials will be required to confirm the internal and external validity of our findings. One large randomized trial (PREDICT-LAA, NCT04180605) has been started in 200 patients [12], as well as a multicentre registry in 100 patients (PRECISE-LAAO, NCT04640051).

While we report only on our first experience with Amulet, FEops also offers simulations for Watchman™ and Watchman FLX™.

## 6. Conclusions

In our first single-centre experience in 15 patients scheduled for LAAO, preprocedural 3D computational modelling by FEops was found to positively impact Amulet size selection in LAAO compared to TOE and CT-based SoC assessment. Operators could consider FEops 3D computational simulation in their preprocedural device size selection.

## Figures and Tables

**Table 1:** Baseline characteristics and clinical indication.

*Baseline characteristics*	*n* = 15

Age (yrs)	77.5 ± 6.5
Male	9 (60.0%)
BMI (kg/m^2^)	26.5 ± 4.1
Congestive heart failure	3 (20.0%)
Hypertension	11 (73.3%)
Age ≥ 75 years	9 (60.0%)
Diabetes mellitus	2 (13.3%)
Previous stroke/TIA	8 (53.3%)
Vascular disease	6 (40.0%)
Permanent AF	11 (73.3%)
Serum creatinine (mg/dl)	1.21 ± 0.63
CHADS-VASc score	4.7 ± 1.0
HAS-BLED score	3.1 ± 0.7

*Indications*	*n* = 15

Cerebral bleeding	3 (20.0%)
Major bleeding	5 (33.3%)
Minor bleeding	3 (20.0%)
Recurrent stroke under OAC	4 (26.7%)

Numbers are mean ± SD or number (%). AF: atrial fibrillation, BMI: body mass index, OAC: oral anticoagulant, TIA: transient ischemic attack.

**Table 2:** Clinical impact in preprocedural sizing.

Patient no.	CT (mm)	Freixa	Decision SoC	Confidence SoC	Decision FEops	Confidence FEops	Implanted
*n* = 7 (46.7%)			Similar decisions SoC and FEops correlating with implanted device				
**1**	16 × 17	22	20	8	20	9	20
**2**	17 × 22	25	22	7	22	9	22
**8**	13 × 19	20	20	8	20	8	20
**10**	27 × 37	34	34	4	34	7	34
**11**	19 × 37	34	31	6	31	8	31
**13**	21 × 36	34	34	6	34	8	34
**14**	19 × 22	25	25	6	25	8	25
Average				6.4 ± 1.4		8.1 ± 0.7	*p*=0.003

*n* = 4 (26.7%)			Different decisions SoC and FEops. FEops correlated with implanted device				
**3**	16 × 18	22	20	8	22	8	22
**5**	19 × 25	28	25	7	22	7	22
**6**	14 × 21	22	22	7	20	7	20
**7**	19 × 26	28	28	7	25	7	25
Average				7.3 ± 0.5		7.3 ± 0.5	*p*=NS

*n* = 2 (13.3%)			Similar decisions SoC and FEops, not correlating with implanted device				
**4**	21 × 31	31	31	7	31	8	28
**12**	22 × 23	28	25	8	25	6	28
Average				7.5		7	*p*=NS

*n* = 1 (6.7%)			Different decisions SoC and FEops, none correlating with implanted device				
**15**	14 × 19	22	22	8	20	7	18
*n* = 1 (6.7%)			Similar decisions SoC and FEops, but no implanted device				
**9**	22 × 31	31	31	7	31	7	NA

CT: computed tomography measurement of landing zone, NS: nonsignificant, SoC: standard of care, and Freixa: sizing based on the sizing table from Freixa et al. [6].

**Table 3:** Procedural characteristics and antithrombotic medication.

	*n* = 15
*Procedural characteristics*	
Fluoroscopy time (min : sec, mean ± SD)	14 : 11 ± 8 : 22
Total DAP (Gycm^2^)	25.1 ± 18.5
Cum Air Kerma (mGy)	265.6 ± 232.7
Amulet size (mm)	
20	3 (20.0%)
22	3 (20.0%)
25	3 (20.0%)
28	1 (6.7%)
31	3 (20.0%)
34	2 (13.3%)

*Antithrombotic medication at discharge*	
Aspirin	3 (20.0%)
Aspirin/Clopidogrel 6 weeks	8 (53.3%)
DOAC/Clopidogrel 6 weeks	3 (20.0%)
VKA	1 (6.7%)

Numbers are mean ± SD or number (%).DAP: dose area product, DOAC: direct anticoagulant, and VKA: vitamin K antagonist.

## Data Availability

The data used to support the study are available within the article.

## Additional Points

*Summary*. Device size selection in left atrial appendage occlusion (LAAO) can be challenging due to a certain degree of variability at both patient and device levels. The clinical impact of a 3D computational modelling (FEops) was evaluated prospectively in the preprocedural Amulet size selection in 15 consecutive patients. FEops-based preprocedural assessment correctly selected the Amulet size in 11 patients (73.3%), compared to 7 patients (46.7%) for the standard of care. FEops correctly induced a change in device size in 4 patients (26.7%). Preprocedural 3D computational modelling impacts size selection. Operators could consider computational modelling in their preprocedural device size selection.
